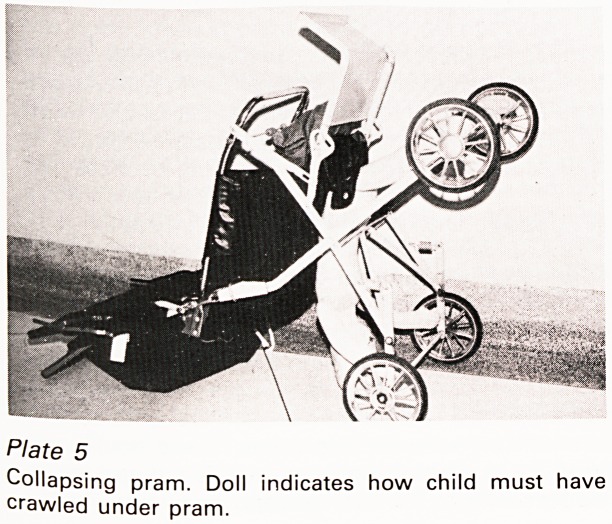# The Postmortem Is Not Dead

**Published:** 1981

**Authors:** N. J. Brown

**Affiliations:** Consultant Pathologist, Southmead Hospital, and Bristol Royal Hospital for Sick Children


### The Postmortem is not Dead

### Presidential Address to Bristol Medico-Chirurgical Society 8th October 1980

**N. J. Brown, MB, FRCP, FRCPath**
Consultant Pathologist, Southmead Hospital, and Bristol Royal Hospital for Sick Children

I am very conscious, ladies and gentlemen, of the honour and responsibility you have conferred on me by asking me to be your president and I shall try to discharge my duties to the best of my ability. I suspect that every president since 1874 has found that his most difficult task is his first one – that of addressing you at the annual general meeting. He has, of course, known for a year that he has to do it and one might imagine that would be long enough to select a subject and prepare a talk, and so it is but it is a year of gathering doubts as to the suitability of the subject chosen and anxiety as to whether it can possibly be of interest to anyone but himself.

Before announcing my subject let me say that over the years I have heard it said disparagingly of many a scientific paper that it was autobiographical, anecdotal or full of phoney statistics. What you are going to hear tonight is all that and worse – but I have several advantages. This is not a scientific paper but a presidential address. Moreover I have a captive audience, I have not had to announce my subject in advance and there will be no questions at the end.

I have chosen to talk to you about postmortem examinations. Contrary to popular belief these do not occupy the whole of a pathologist's time but they are an important part of his work and I would like to pass on to you some of my thoughts about what I have learned and what we can all still learn from examination of the human body after death.

### ARMY PATHOLOGY

My story begins in 1945 in the town of Bari on the Adriatic coast of Italy. Serving in the Royal Army Medical Corps I had, throughout the Italian campaign been medical officer to a regiment of the Royal Artillery having come with them from the Middle East as part of Montgomery's Eighth Army. When the war in Europe ended and the regiment broke up I had applied for training as a pathologist, and after a rather unnerving interview with Colonel (later Professor) H. L. Sheehan, then Director of Pathology for the Central Mediterranean Area, had been accepted and started training in hospitals in Rome and then Loreto. When I had received about half the training a Senior House Officer would nowadays be given, I found myself, on returning to Italy after my first leave in England for nearly three years, posted to a hospital in Bari.

I was no stranger to this town, having passed through it two years previously with the gunners, and naturally assumed that I was to continue my apprenticeship. To my horror, however, I found that I was no trainee but the only pathologist to a 1200 bedded hospital, full of patients, two previous pathologists having been invalided home in quick succession with amoebic dysentery. Next day the only qualified laboratory technician left for home and I was presented with somewhat the sort of problem any SHO would have if left to run the Bristol Royal Infirmary laboratory on his own.

I had been in the army long enough to be not unacustomed to what appeared to be ghastly military blunders – the gunners had a word for them, SAMBUs, Self-adjusting military botch-ups (or something like that) – and I had the simple soldier's faith that somebody, if only General Alexander, knew what was going on and I expected it would put itself right. So it did eventually but not for several months during which time I toiled from dawn to dusk doing what I could to deal with investigations which despite being kept to a praiseworthy minimum by sympathetic colleagues, nevertheless were numerous and varied. During this time I lived in dread of being asked to carry out a postmortem examination; it was not that I did not know how but rather that I could not spare the time away from the multitude of blood counts, urines, swabs and other clinical investigations that took up all my waking hours. Inevitably the day came, a nursing orderly was detailed to assist me, and I embarked on my first necropsy. It was a case of death from multiple injuries. I wrote a report, made a copy and proudly wrote on it 'PM No.1'. Always having been a bit of a squirrel I kept it and so started a habit whereby I have kept a copy of the report on every postmortem examination I have carried out. I now have nearly 17,000 of them. These are what I want to tell you about but first I must finish my story and explain when, where and why these examinations came to be done.

I was, in time, rescued from Bari by the intervention of Colonel (later Professor) George Cunningham, who had succeeded Sheehan, and was sent off for further training in Naples. I became what the army described as a graded specialist and then spent a year as pathologist to a small army hospital in Trieste. There I not only became adept at the diagnosis of typhoid, diphtheria, malaria, dysentery and syphilis but also made a valuable liaison with the professor of pathology at the civilian medical school. From Vienna, schooled in the traditions of Rokitansky and persisting in abortive attempts to converse in what he said was latin, the true international language of cultured doctors, he not only allowed me to watch the postmortem examinations in his department but also gave valuable assistance with histological problems. When I returned to England in 1947 I had carried out nearly 50 postmortems, mostly cases of accident or crime.

### UNIVERSITY PATHOLOGY

After leaving the army I worked for several years in the Pathology Department of the University of Bristol which was then housed in Canynge Hall in Whiteladies Road, an old Victorian building which before conversion to laboratories had been a hall of residence and before that a hotel. It still retained many of its original features such as ornamental ceilings and fireplaces and its very quaintness endeared it to all who worked there. The department was ruled over by Professor Tom Hewer to whom I owe a tremendous personal debt of gratitude for a thorough disciplined training in histopathology. His insistence on scrupulous cleanliness and meticulous technique will never be forgotten, nor will his scorn of loose thinking, unwarranted deductions or untidy presentations. All postmortem findings had to be paraded before the whole department on Wednesday afternoons and nothing was filed until he had approved it. Attendance at clinico-pathological conferences, bone tumour registry, journal club and other meetings, mostly held in the evening after a full day's work was virtually compulsory. His enthusiasm for pathology was infectious and throughout it all there was the constant emphasis on correlation of pathological findings with clinical features. My years at Canynge Hall were probably the most valuable in my whole professional life.

During that time the question arose of obtaining a higher qualification. In those days before the foundation of the Royal College of Pathologists the option was to take either an MD by thesis or MRCP by examination. Original thinking never having been my strong point, I opted for the latter and joined in my spare time, a self-help group known affectionately as the 'cardio-respiratory harriers' whose members in turn attempted the hurdle of the MRCP examination every three months. All were ex-servicemen trying to make up for five years or so 'lost' in the forces by organising ward rounds and tutorials for their mutual edification. I have a feeling that we may have learned more in this way than people do nowadays from more sophisticated courses of instruction. After I will not say how many attempts, the scepticism of the censors of the Royal College of Physicians was overcome and I achieved my objective but, more important, I had received a good grounding in clinical medicine which I shall never regret. I had of course gained a good deal of clinical experience in the army since even as a so-called pathologist one was always allocated some general duties. In Trieste in fact I had been designated paediatrician to the families of senior officers who were beginning to appear in Italy as the forces moved on to a peace-time basis. This experience turned me against upper-class paediatrics for life but was probably less traumatic to me than was the lot of the army-trained psychiatrist who was detailed to be their obstetrician!

### SOUTHMEAD HOSPITAL

In 1949 as a Lecturer in the University Pathology Department I was sent to Southmead Hospital on loan to help to organise a new histopathology service and in 1951 was fortunate in obtaining a newly created Consultant post. In the field of surgical histology I had the immense benefit of the help of Dr. Frank Lewis on whose very wide experience I leaned heavily and whose freely given advice was invaluable. All the postmortems however became the responsibility of myself and a series of young Demonstrators from the University who changed every one or two years. Over the next twenty years under this system man and boy (or often man and girl) carried out some 12,000 postmortems about a quarter of which were on stillborn or newborn infants. This works out at 600-700 per year or an average of two per day including Saturdays.

We worked in cramped conditions in the early days but thoroughly enjoyed our work, often experimenting with new and sometimes unorthodox techniques. We cut and examined endless microscopical sections and collected and mounted innumerable interesting specimens, creating especially a comprehensive museum of perinatal pathology. I often compared myself during this period to Haydn in the court of Prince Esterhazy. Just as he had his own personal orchestra and could experiment with composition to his heart's delight so did I have my enthusiastic band of assistants and technicians with complete freedom to investigate my cases in any way I wished. I was particularly appreciative of the high quality of the young demonstrators who came from the university in those days. They were allowed to stay with me long enough to establish a good working relationship; almost all went on to become consultants in pathology, and one a distinguished professor. Let me hasten to add that I claim no responsibility for this; they would have done so anyhow, no matter where they worked, they were that sort of people.

### RURAL PATHOLOGY

Our activities spread. We took over the post-mortems at Hortham and Brentry Hospitals and for a time at Stoke Park Hospital. We extended our field backward in time to include aborted fœtuses and even placentas, a rather unusual occupation in those days. Remarks were heard about the 'Southmead Dissecting Corporation'. Then I was asked by the Coroner for South Gloucestershire if I would take on his postmortem examinations which had hitherto been done by general practitioners.

This was as picturesque a bit of pathology as it is possible to imagine. The examinations were carried out in little mortuaries dotted about all over the county (Plate 1). At Staple Hill the mortuary, the most modern and the only one with a refrigerator was in a corner of the council yard among the kerbstones, lamp-posts and rubbish bins. The Kingswood mortuary was part of a public lavatory. At Yate one found the mortuary in an apple orchard down a long muddy lane; the examination table was on wheels and to avoid chasing the body around the room the custom was for the attending policeman to jam the table against the wall with his foot. At Berkeley and Thornbury there were mortuaries in the cottage hospitals. Filton mortuary, now demolished, was in a field exactly opposite the end of the runway of the aerodrome and its crumbling structure trembled every time a plane took off. The prettiest mortuary was at Wotton-under-Edge, nestling at the foot of the Cotswolds; this was the only one to be manned by a mortuary keeper, an ex-naval man usually to be found in the hostelry next door. Elsewhere it was necessary to take one's own assistant.

The necropsies were attended by the local policeman who also opened up the building and switched on whatever light and heat was available. There was very good clinical correlation since the police officer usually knew the deceased and often had a store of irrelevant but interesting anecdotes. Amusing incidents sometimes occurred particularly in relation to identification. One body was confidently but wrongly identified as that of a tramp who on his next visit to the village was shown an account of the inquest into his death. On reporting to the police station that he had just read about his death in the newspaper, he is said to have received the phlegmatic reply 'Oh yes Sir, and where might you have come from now, then?' A dear old countryman, asked to make a formal identification of his dead wife, found himself unable to be certain from her face that it was her. An impasse seemed to have been reached when he suddenly produced the solution. 'Let me see her feet', he said and after one quick glance pronounced that it was undoubtedly his wife and could be none other. I went back and studied those feet carefully afterwards but I still do not know how he did it.

This rural activity, fascinating as it was, took up more time than could be spared, often in the early morning or late evening and I eventually persuaded the Coroner to have all his bodies sent to Southmead which by then had a splendid new mortuary. My biggest regret was losing the contact with the local policeman. From now on it was decreed that he would write a short history on a luggage label and tie it to the subject's toe. This was a poor substitute for many hours of pleasant conversation.

### THE LAST DECADE

On the death of Dr. A. D. Fraser who for very many years had been responsible for the postmortems for the City of Bristol Coroner it became necessary to devise a way of covering the work at the Bristol City Mortuary. A rota system was evolved in which I participated. The major turning point however came in 1971 when Dr. Colin Tribe was appointed to Southmead Hospital and additional junior staff

*Plate 1*
Rural mortuaries: (a) Filton; (b) Kingswood; (c) Yate; (d) Wotton-under-Edge.

*Plate 2*
Mortuary technicians: (a) Frank Holland; (b) Douglas Wakefield.

became available. This enabled me to devote almost the whole of my time to what had always interested me most, namely the pathology of children and particularly the newborn, and I readily accepted the offer to take on the histopathology for the Bristol Royal Hospital for Sick Children and later the new Bristol Maternity Hospital while continuing to be responsible for the paediatric pathology at Southmead; some coroner's work also continued although in diminishing amount in recent years.

I will now cease boring you with the story of my life which I have introduced merely to show how the mass of material which I now propose to present to you was collected. Before turning to this however I would like to pay tribute to the men without whose help all this work would not have been possible – the Mortuary Technicians. They are the salt of the earth. There is something about mortuary work which attracts or breeds men of great character. Unfailingly helpful and eternally cheerful the good mortuary man is a joy to work with. Perhaps he shares the pathologist's enthusiasm for solving puzzles and seeking the truth but, whatever the reason, the job seems to become absorbing and fascinating to him and very rarely does he wish to change it for another. Some of you will remember the two whose portraits I now show you (Plate 2), both from Southmead and both, sadly, now departed this life – Frank Holland, a true son of Bristol if ever there was; he died in the hospital he had served for forty years and was laid to rest in his own mortuary – and Douglas Wakefield, the life and soul of so many hospital activities; coming to us from his previous trade as a butcher his special skills were invaluable but I doubt if he ever came to terms with our greater interest in the offal than the joints.

### THE MATERIAL

My 16,800 reports of personally conducted postmortem examinations to date form a heap four feet high and six feet wide. For the past twelve months I have been browsing through them with some, at least to me, interesting results. The exercise has confirmed that one's memory plays the most extraordinary tricks. Conditions which I would say I had never seen are recorded in my own handwriting, completely forgotten, while other findings which I would regard as relatively common prove to have been rarely encountered. There are cases which I have recounted to generations of trainees and students where the present version bears little relation to what actually occurred. I suppose this must happen to other people but it is an insight into human fallibility and a strong argument for keeping, and referring to, accurate records.

**Table 1**

**Number of Postmortem Examinations by Age Groups**

| | |
|---|---|
| Adults | 12,218 |
| Children (1 month to 15 years) | 1,011 |
| Babies (under 1 month) | 3,571 |
| | 16,800 |

**Table 2**

**Number of Postmortem Examinations by Cause of Death**

| | |
|---|---|
| 'Natural Causes' | 15,395 |
| Accidental death | 922 |
| Suicide | 473 |
| Crime | 10 |
| | 16,800 |

Table 1 shows the distribution of the cases by age groups and underlines the large number of infants and children. Table 2 gives broad groupings of the causes of death. The number of deaths resulting from crime is somewhat understated as criminal proceedings arose from a number of cases classified as accidental, mainly road accidents, but the fact remains that I have never been much interested in crime and would prefer such cases to be dealt with by experts in the field. I am certainly in no position to regale you with stories of scientific detection. One is fairly regularly called out, quite properly, by the police to scenes of death where suspicions have been aroused but the great majority of these cases prove to have other explanations.

I will now review some aspects of the material under the headings of accidents, sudden deaths and suicide followed by some remarks on the newborn and some philosophical conclusions about postmortem examinations in general.

**Table 3**

**Causes of Accidental Death in Adults**

| | |
|---|---|
| Road and train (11) | 473 |
| Falls | 93 |
| Drowning | 63 |
| Burns and fires | 45 |
| Poisoning | 43 |
| At work | 38 |
| During recreation | 18 |
| Electrocution | 5 |
| Other | 6 |
| | 784 |

**Table 4**

**Causes of Accidental Death in Children**

| | |
|---|---|
| Road and railway (1) | 64 |
| Drowning | 16 |
| Burns and scalds | 15 |
| Mishaps during play | 14 |
| Falls | 11 |
| Poisoning | 5 |
| Asphyxiation in cot or pram | 5 |
| Crushing | 4 |
| Electrocution | 4 |
| | 138 |

### ACCIDENTS TO ADULTS

Table 3 shows the principal causes of accidental death in adults. It will be noted that three quarters are due to road accidents, falls and drowning. The falls referred to here are from roofs, ladders and so forth and do not include the very common deaths following fractured femurs in the elderly, the practice being to regard these injuries as pathological fractures associated with osteoporosis and the deaths therefore classifiable as due to natural causes. The majority of the accidental poisonings were caused by carbon monoxide from defective gas appliances, car exhausts or burning naked flames such as oil heaters in confined spaces like caravans. Accidents at work usually involved machinery. Recreational accidents occurred during obviously dangerous activities such as climbing and motor racing but three deaths resulted from a slipping jack during DIY car repairs and even gardening was not without risk: one unfortunate woman tugged so hard on a recalcitrant weed that it snapped causing her to fall over backwards and impale herself on an iron spike hidden in the undergrowth. Electrocution, although often suspected, was a quite uncommon cause of death.

### FATAL ACCIDENTS IN CHILDREN

Accidental deaths in children, shown in Table 4 follow a different pattern. As might be expected nearly half are due to road accidents and deaths from drowning and burns are numerous. The fatal mishaps during play form an interesting group. Some were due to recognised causes such as asphyxiation by inhaling peas from a pea-shooter but other were quite unforeseeable. A lad tripped and became impaled on a javelin during sports practice; a girl pierced the roof of her pharynx with a knitting needle while emulating a sword-swallower she had seen on television, and died of meningitis; another little girl dressed up in her grannie's clothes and shoes, tripped on the stairs, got her fancy dress entangled in the bannisters and died of suffocation.

Of the five fatal poisonings, two were from carbon monoxide, one from paraquat due to weed-killer in an unlabelled container being mistaken for lemonade, one was from digitalis overdose due to a prescription error and one from the experimental ingestion of Ponderax tablets left on a kitchen table.

Some crushing injuries resulted from children being in places where they should not have been. One lad climbed into the belfry of Westbury parish church while the bells were being rung and became trapped by a bell accross his chest.

Of the asphyxiations in cot or pram, three were caused by devices designed to stop the child falling out; this is ironical since I have never seen a death from a child falling out of bed. One such case which received wide publicity involved a child who became entangled in such a harness (Plate 3). At the inquest despite a demonstration to the jury with a life-size doll of exactly how this could happen, the manufacturer maintained that the apparatus was not unsafe if used according to the instructions. These were complicated and involved adjustment of tensions in various straps and in any case had not been available to the unfortunate grandmother who had borrowed the device from someone else. The court took the view that if these instructions were so vitally important they should

*Plate 3*
'Safety' harness. Demonstration with a doll to show how a child could get a loose harness around the neck.

*Plate 4*
Collapsing pram. Doll indicates position in which child was found.

*Plate 5*
Collapsing pram. Doll indicates how child must have crawled under pram.

be firmly attached to the apparatus but this was stated to be impossible. I believe the device was later withdrawn from sale after pressure from a number of quarters but no doubt many of them remain in circulation, some no doubt lacking the all-important rules.

A rather different attitude was taken by another manufacturer, this time of a folding pram, which, one Christmas Eve, collapsed, crushing a little girl to death like a giant mouse trap (Plate 4). It had safety rings and for a long time we could not see how the accident occurred until a police officer worked out what could be the only solution; the child must have crawled over the axles under the pram causing it to tip on to its handle so that the rings slid down under their own weight releasing the springs. Experiments with a doll confirmed this (Plate 5). So did the manufacturer at the inquest for, unknown to us, there had been a previous fatality which occurred in exactly that way. After that incident all the prams had been withdrawn from shops until a modification had been made but, as he pointed out, prams have a long life and pass through many hands and he estimated that there must be many thousands of the original models still in use and untraceable. One can sympathise with his distress: what designer could ever have visualised a child getting itself into such an unlikely situation?

### SUDDEN DEATH

The great majority of Coroner's cases are examples of sudden death from natural causes of which, of course, the most common are coronary artery disease, myocardial infarction and other vascular lesions such as ruptured aneurysms. There are many other causes some of which are more often seen than many clinicians might suppose. I have, for example, been impressed by the frequency of acute left ventricular failure with pulmonary oedema associated with calcific disease of the aortic valve. Many of these individuals have never consulted a doctor and for the same reason I would not be surprised if cardiomyopathy in young people is more often seen by pathologists than any other doctors since their heart disease is usually quite unsuspected.

I have always been interested in what individuals were actually doing when sudden death overtook them. In many cases the only history available is 'collapsed and died' or 'died suddenly at home' or something similar but in going through my reports I have found about 1600 cases where the circumstances at the time of

**Table 5**

**Circumstances of Sudden Collapse and Death**

| | |
|---|---|
| In the street | 301 |
| During recreation | 255 |
| At work | 222 |
| In bathroom or toilet | 150 |
| Gardening and home maintainance | 120 |
| Sitting in chair | 118 |
| In motor vehicle | 77 |
| During stress or exertion | 77 |
| At meal or in kitchen | 69 |
| In bed | 47 |
| Other *(see Table 7)* | 196 |
| | 1632 |

sudden death are recorded in detail and these may be worth noting if only to help you to avoid the situations conducive to sudden demise.

A glance at Table 5 will show you that you will find this difficult. The most dangerous place to be is in the street and to indulge in recreation (see below) is inviting trouble. Work carries a high risk and one should think twice about visiting the toilet, doing any gardening or sitting in a chair. These six occupations account for more than half the cases. Of the deaths in motor vehicles most involved the driver: several were taking driving tests and in at least one instance it was the examiner who collapsed. 'Stress and exertion' include interviews, pushing cars, sweeping snow and being involved in arguments and commotions; this group is smaller than one might have expected. A word of explanation is necessary in regard to sudden death in bed. In constabulary parlance there is a subtle distinction between 'died in bed', 'found dead in bed', neither of which are included here, and 'collapsed and died in bed' which is usually taken to mean that the deceased was indulging in some activity at the time.

### SUDDEN DEATH DURING RECREATION

The relative risks of various forms of recreation are indicated in Table 6. The sports and games involved, some rather surprising, were dancing (7), bingo (6), bowls (5), cards (4), skittles (4), golf (3), riding, shooting, table tennis, hockey, badminton, croquet, darts, tennis, keep-fit (3), football, rugby, skating, jogging (2) and squash. It is perhaps noteworthy that I have never met anyone who died while playing cricket. There were however two deaths at cricket matches among those watching

**Table 6**

**Examples of Sudden Death during Recreation**

| | |
|---|---|
| Sports and games, participating | 48 |
| In public houses | 30 |
| Watching television | 22 |
| Walking | 17 |
| Watching games | 13 |
| In clubs | 11 |
| Pursuing hobbies | 10 |
| At cinema | 8 |
| | 159 |

**Table 7**

**Some Other Circumstaces Surrounding Sudden Death**

| | |
|---|---|
| Seeing doctors | 44 |
| In shops | 43 |
| On buses | 39 |
| In church | 27 |
| Railway stations and trains | 20 |
| On ships | 7 |
| Unlikely | 16 |
| | 196 |

sport. The other spectators in that group were football supporters (more often Bristol City than Bristol Rovers). Those pursuing hobbies did not on the whole appear to be stressfully occupied; several were fishing or tending pigeons.

### OTHER CIRCUMSTANCES

Table 7 analyses further the cases labelled 'other' in Table 5. That some people collapse while seeing doctors is to be expected. Shops should be visited with caution. Bristol buses appear to have something to answer for and it must not be forgotten that these were the people who actually caught a bus: many of those previously referred to who died in the street were waiting at bus-stops. Of the deaths in church, five were at funerals, one at a wedding and one was the preacher. The circumstances labelled 'unlikely' included shaving, needlework and sitting in public libraries.

### SUDDEN INFANT DEATHS

To adopt a more serious note, I have dealt with 315 of these distressing cases where an apparently healthy infant, almost always less than eight months old is found dead in its cot or pram usually having apparently died peacefully during sleep. Postmortem examination reveals either no disease or what appears to be minor disease, often respiratory tract infection. How much pathology one finds depends on how hard one looks and, to me, as a paediatric pathologist, the cases seem to demand intensive investigation. The cause of these deaths has baffled investigators all over the world but there is perhaps now the glimmer of a hope of explanation. It seems that certain infants suffer from some inherent physiological defect, probably concerned with sleep and respiration, so that they are living on a knife-edge of liability to sudden death if a number of different precipitating factors come into operation. I anticipate that the underlying defect will be discovered in the not too distant future by the physiologists; what the pathologist can do by applying not only histological but chemical, bacteriological, virological and even more unorthodox investigations, is to identify some of the precipitating factors. More of these are being found all the time but it is important to recognise them for what they are, merely the final insults, not single new final solutions to a complicated problem. For example, we carried out screening tests for common drugs on samples from some of these babies. Of 125 tests, no drugs were found in 93 but benzodiazopines were found 8 times, barbiturates 5 times, ethanol 3 times and salicylates once; unidentified probably toxic compounds were found in another 15. The drugs were present only in small amounts and no-one is suggesting the infants were deliberately poisoned. Some may have been passed to the baby through mother's milk. However they got there, one cannot help wondering whether they might have made the baby sleep more heavily, to its detriment but on the other hand it could merely be a reflection of social custom in that maybe every night ten per cent or so of all babies in Bristol are given small doses of grannie's sleeping tablets or a sip of alcohol to get them to sleep; in the absence of controls we shall never know.

It is terribly important when these deaths occur that someone should offer to try to give counsel and support to the parents. Not all parents will immediately accept the offer but many are completely bewildered and desperate for informed advice. Sometimes they will ask to see the pathologist as, strange to say, he may be the only doctor they feel they can trust and I do not think he should refuse. In any event, as of course in many other types of case, he should make his findings known as soon as possible to the practitioner who is going to talk to the bereaved relatives, be he paediatrician, family doctor or whatever.

**Table 8**

**Methods of Suicides**

| | *Men* | *Women* |
|---|---|---|
| Overdose of drugs | 56 | 114 |
| Gassing | 60 | 49 |
| Hanging | 58 | 10 |
| Jumping from height | 36 | 9 |
| Drowning | 16 | 10 |
| Shooting | 19 | 1 |
| Car exhaust fumes | 12 | 1 |
| Self-wounding | 8 | 1 |
| Ingestion of poisons | 4 | 1 |
| Miscellaneous | 3 | 5 |
| | 272 | 201 |
| | 473 | |

### SUICIDE

Table 8 shows the methods used in 473 suicides. It will be observed that the pattern differs between men and women, the latter showing an overwhelming preference for taking overdoses of drugs while men opt for hanging, shooting or wounding themselves, jumping from heights such as the Clifton suspension bridge or connecting hoses from the exhaust pipes to the interior of cars. The only woman to adopt the last method was probably not a true suicide but died by mistake. She had previously taken an overdose of drugs and on another occasion threatened to jump off a building in circumstances where she was certain to be rescued. She connected up her car's exhaust pipe not in secret in the dead of night but in full view in broad daylight in a busy public car park. The great British Public however, being what it is, did not presume to interfere.

The youngest suicides were a boy aged 12 who, embarrassed by bed-wetting, took an overdose of the Imipramine with which he was being treated, and a girl aged 14, crossed in love, who took sleeping tablets. Of the poisonings other than drug overdoses three were by cyanide. One man's mouth was stuffed with solid sodium cyanide which he had obtained from his work in an electroplating shop; I shudder to think what might have happened had someone attempted mouth-to-mouth resuscitation! There were several cases of

**Table 9**

**Drugs used in Suicidal Overdoses**

| | |
|---|---|
| Barbiturates | 89 |
| Antidepressants and tranquillizers | 48 |
| Non-barbiturate hypnotics | 33 |
| Analgesics | 14 |
| Other | 4 |
| | 187 |

self-electrocution, all men, involving elaborate preparations of wiring and switchgear and cool calculating determination but the most bizarre method of self-destruction was adopted by a man who lay down beneath a hopper used for loading lorries with sand, actuated the mechanism by previously contrived remote control and buried himself so deep that he was only discovered next day when disgruntled workmen presuming a fault in the hopper were obliged to clear away the mountain of sand by hand.

In view of the many cases of drug overdose it may be of interest to record what drugs were used (Table 9). The survey extends over thirty years and fashions in drugs change, barbiturates for instance are less commonly prescribed nowadays than formerly. In not all the cases was the drug known and many subjects took more than one drug. I am constantly amazed at the vast quantities of drugs brought in by the police from people's homes. There must be something wrong with a system which allows so many individuals to accumulate, from legitimate prescriptions, enough soporific material to send a large part of the population of Bristol to sleep.

### BABIES

The collection includes my own records of 3571 perinatal deaths of which 1978 were stillbirths and 902 were prematurely born infants. Many hundreds more were examined under my supervision by the many acolytes into whom I have tried over the years to instill the same interest in the problems of the newborn that I myself developed.

When I was first appointed to Southmead Hospital I was taken aside by the late Professor Victor Neale and given fatherly advice along these lines. 'You are young and keen but you cannot investigate everything. Forget about atheroma and all those conditions that any pathologist in the land can look into. Here you have a golden opportunity; you have the second biggest maternity unit in the country. Concentrate on the material you have and others have not. Put all your spare resources into finding out why so many babies die. It will be well worthwhile.' How right he was. In those days very few postmortems were done on babies and to regard it as important was deemed to be rather eccentric. To label an infant as 'stillborn' was felt to be sufficient and few pathologists made much attempt to find out why. The whole field of perinatal death was wide open for exploration. Very little was written down. New observations could be made without complicated apparatus by the simple use of hands and eyes. One fumbled along, correlating pathological findings with clinical observations at fortnightly meetings with obstetricians and paediatricians also instituted by Victor Neale and still continuing – although monthly now as there are far less deaths. Events like the foundation of the Paediatric Pathology Society and the publication of Edith Potter's and Agnes MacGregor's classic books brought isolated workers together and pooled knowledge. Concepts such as congenital infection, placental insufficiency, postmaturity and intra-uterine malnutrition were gradually defined. Iatrogenetic conditions such as retrolental fibroplasia and kernicterus in premature infants due to over-use of oxygen were recognised. The roles of haemolytic disease, cystic fibrosis and maternal diabetes became more clearly understood. There were new ideas about congenital malformations and some aetiological factors were identified. Perinatal deaths have been greatly reduced over the last thirty years and there is no doubt in my mind that the knowledge gained from examination of the dead babies is such that they did not die in vain. Moreover the exercise is not over; there is a great deal still to learn and baby deaths are still more frequent than they need be. The educative value of the perinatal postmortem is as great as ever.

### THE POSTMORTEM EXAMINATION

Some people now say that the postmortem is an outmoded exercise and in the light of modern scientific advances no longer has the value it once had. In my opinion, despite being now called a histopathologist whereas I used to be a morbid anatomist, this is dangerous rubbish. In this address I have talked mainly about examinations carried out to determine the cause of death but there are other reasons for doing postmortems. They are invaluable as a means of teaching pathology and they provide a constant check on an institution's standard of accuracy of clinical diagnosis by providing an opportunity to correlate clinical deductions with what is really there. Without this 'audit' there would be a real danger of the clinician evolving a totally imaginary system of pathology as happened for 1500 years during the middle ages when dissections of the human body were not carried out. Dr. Stewart Smith, writing in our Journal nearly a quarter of a century ago (Smith 1956), asserted that it was seldom that postmortem examination failed either to modify or to change the diagnosis. I think that may still be true today.

*To the Clinician I would like to say:*

Ask for a postmortem even if you feel sure of the diagnosis. Don't go back to the middle ages.

Insist that the examination is done properly, either carried out personally or supervised by an experienced pathologist, not left as a chore for the most junior member of the department.

Go and see it. It is a consultation over a patient, even though dead, not a laboratory examination like asking for a haemoglobin estimation.

*To any young pathologist I would like to say:*

Remember that to dissect a human body is a privilege accorded to very few and conduct yourself accordingly.

Never gloat if you find something the clinicians missed. You have an enormous advantage over them and if you have any experience of clinical practice you should know how difficult it is.

Welcome an audience and make things interesting for them by cultivating a bit of dignified showmanship and a flexible technique. A busy physician interested mainly in the heart will never come again if you keep him hopping from one foot to the other while you make a laborious dissection of the bile ducts.

And *finally,* of course – keep all you reports so that when you are given an opportunity you can bore an audience to death with your reminiscences.

**REFERENCES**

MacGREGOR, A. R. (1960). Pathology of Infancy and Childhood. Livingstone, Edinburgh and London.

POTTER, E. L. (1952). Pathology of the Fetus and the Newborn. 1st edition. Year Book Publishers Inc. Chicago.

SMITH, G.S. (1956). A morbid view of the over-65s. *Medical Journal of the South-West,* **71,** 44-54.

## Figures and Tables

**Plate 1 f1:**
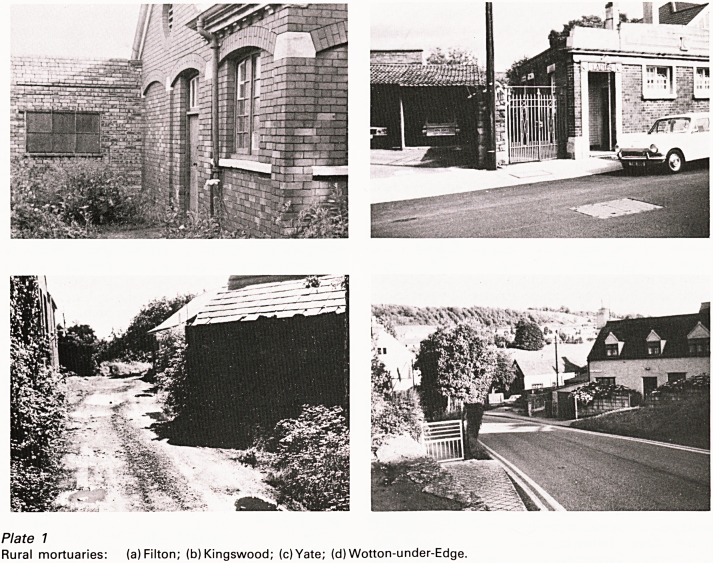


**Plate 2 f2:**
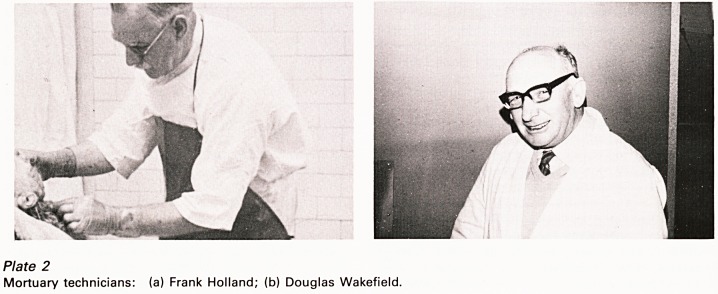


**Plate 3 f3:**
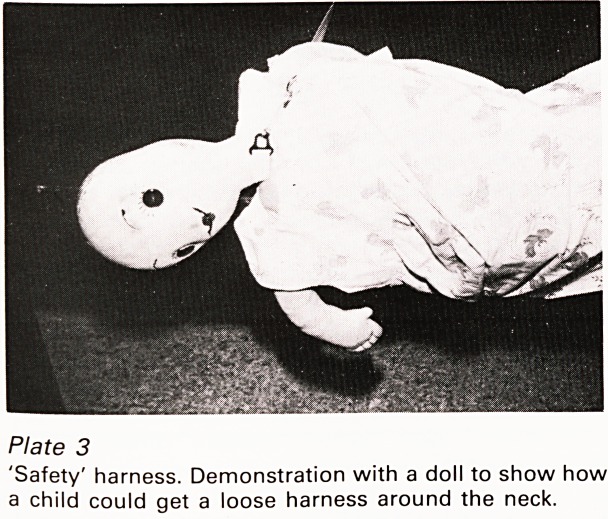


**Plate 4 f4:**
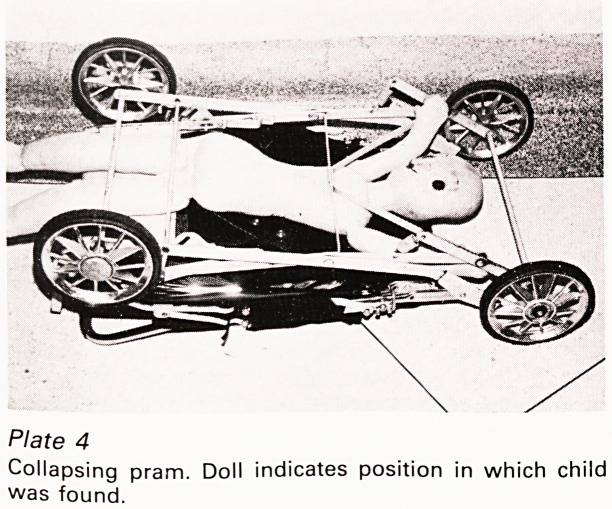


**Plate 5 f5:**